# In-Hospital Mortality From Cerebrovascular Disease

**DOI:** 10.7759/cureus.8652

**Published:** 2020-06-16

**Authors:** Otto Jesus Hernandez Fustes, Carlos Arteaga Rodriguez, Olga Judith Hernandez Fustes

**Affiliations:** 1 Neurology, Complexo Hospital de Clínicas - Universidade Federal do Paraná (CHC- UFPR), Curitiba, BRA; 2 Neurology, Universidade Positivo, Curitiba, BRA; 3 Neurophysiology, Clinica Neurológica das Américas, Curitiba, BRA

**Keywords:** mortality, stroke, cerebral ischemia, hemorrhagic stroke

## Abstract

Introduction

Cerebrovascular disease (CVD) is the second most common cause of death. Despite the advances made in recent years with the introduction of specific treatment units and thrombolytics, CVD remains the leading cause of neurological hospitalization and adult disability.

Objective

Our objective is to determine the frequency and causes of early mortality, during hospitalization, of patients with acute CVD.

Methods

We conducted a retrospective, descriptive study of 704 patients treated for acute CVD at the Neurology Service of the Hospital in Curitiba, Brazil, over a period of three years, to whom the CVD Program protocol was applied. We checked the conditions at hospital discharge, obtaining the mortality rate and its causes.

Results

We studied 463 men and 241 women, over 14 years of age with an average of 64 years; 57 patients died. Of the 614 with ischemic CVD, nine males and four females died, establishing a mortality rate of 1.9%. Of the 90 patients with hemorrhagic CVD, 44 died: 26 male and 18 female. The main causes of death were arrhythmias, pneumonia with acute respiratory failure, acute myocardial infarction, and multiple organ failure.

Conclusion

We found no relationship between mortality and specific risk factors, except for age over 65 years. The low rate of deaths obtained in ischemic stroke reflects the multidisciplinary work involved in caring for patients with cerebrovascular disease in our center, which allows us to obtain results as low in mortality as those described in the literature.

## Introduction

In 2013, stroke was the second most common cause of death (11.8% of all deaths) worldwide after ischemic heart disease and the third most common cause of disability [1]. The Global Burden of Disease 2010 showed that stroke mortality rates decreased significantly in both high- and low- and middle-income countries with a lower reduction in the poorest countries [2-3].

Stroke is a major public health problem, however, there is little focus on the control of risk factors, organizing medical care, and funding of research in this field. Since 2005, the Brazilian Ministry of Health has incremented actions aiming at improving the definition of causes of death in the country, implementing standardized instruments for the investigation of deaths, training teams of death surveillance, and establishing goals and quality indicators of the Mortality Information System [4].

The present study aims to find out about in-hospital mortality from cerebrovascular disease and its causes in our country.

## Materials and methods

With the help of the data center of the Data Processing Center of Hospital Universitário Cajurú, records were obtained of patients admitted with a diagnosis of acute cerebrovascular disease in the hospital in three years. Based on this information, we performed an active search of medical records and their CVD protocol, obtaining demographic data, CVD classification, clinical evolution, and cause of death. The subtypes of stroke were defined using universally accepted criteria: ischemic stroke and intracerebral hemorrhage on the basis of computed tomography (CT) scan findings and subarachnoid hemorrhage on the basis of CT scan and cerebrospinal fluid (CSF) findings. The data obtained were tabulated and the frequency of mortality and its causes by sex and CVD classification (ischemic or hemorrhagic) were calculated. The study was waived by the Research Ethics Committee because it exclusively uses databases of public domain, without nominal identification. The ethical principles contained in the Resolution of the National Health Council (CNS) n. 466 of December 12, 2012, were observed.

## Results

We analyzed 704 patients (463 men and 241 women) over 14 years of age, with an average of 64 years of age; of these, 57 patients died. We had 614 with ischemic CVD (406 male and 208 women), and 13 died during hospitalization (9 male and 4 female), establishing a 1.9% mortality rate. Ninety had hemorrhagic CVD, of which 44 died (26 men and 18 women) (Table 1).

**Table 1:** Mortality for CVD and gender CVD: cerebrovascular disease

	Ischemic CVD				Hemorrhagic CVD		
Gender		No. Cases	Deaths	%	No. Cases	Deaths	%
Male		406	9	2.21	57	26	45.61
Female		208	4	1.92	33	18	54.54
TOTAL		614	13	2.11	90	44	48.88

The main causes of death were multiple organ failure, pneumonia with acute respiratory failure, and arrhythmia, in that order. (Table 2)

**Table 2:** Causes of death

Ischemic CVD				Hemorrhagic CVD			Total	
Causes	male	female	total	male	female	total		
Multiple organ failure	3	2	5	11	4	17	22	
Pneumonia with acute respiratory failure	2	1	3	5	6	11	14	
Acute myocardial infarction	2		2	5	3	8	12	
Arrhythmias	1	1	2	3	4	7	9	
Others	1		1	2	1	3	4	
TOTAL	9	4	13	26	18	44	57	

## Discussion

From international population studies, we can infer that in Curitiba, there are seven to eight strokes per day. In the United States, about 500.000 people suffer a stroke each year and of these, approximately 150.000 will die. Cerebrovascular disease consumes approximately 40 billion dollars a year in the United States with direct and indirect costs. These figures demonstrate that cerebrovascular disease has a major impact on population health and economic policy in countries [2-3,5].

Mortality from the first cerebral infarction was studied by Baptista et al., who analyzed 3,362 patients from the Lausanne Cerebral Infarction Registry, determined overall mortality of 4.8%, and the classification into hemorrhagic and ischemic revealed mortality of 14.4% and 3.7%, respectively [6]. Comparing groups of patients 75 years of age or older, with and without atrial fibrillation, Kaarisalo and colleagues demonstrated that mortality from cerebrovascular disease is higher in the population where atrial fibrillation is present, both in the 28-day period (33.9% x 28.1%) and after one year (52.7% x 43.0%) [7]. In another study, involving patients aged 35 to 74 years old, Kaarisalo et al. also faced higher mortality in the group of patients with atrial fibrillation [8].

A socioeconomic study of mortality from cerebral infarction was developed by Kunst et al., noting that in all countries studied, mortality from cerebral infarction was higher in the group of manual workers than in the non-manual group; possibly due to exposure to risk factors and quality of medical care [9].

Several studies have correlated the level of cholesterol and the risk of dying from CVD with controversial results [10-11]. When properly treated, systemic arterial hypertension, being one of the most frequent risk factors, reveals a 36% reduction in mortality and a 35% reduction in CVD morbidity [12].

Jorgensen et al. studied CVD mortality in patients with diabetes and non-diabetics, demonstrating that mortality in the diabetic group (24%) is higher than in the non-diabetic group (17%) and that diabetes is associated with a risk relative to 1.8 to die from cerebral infarction. In the same study, the authors observed that the mortality of non-diabetic patients is dependent on the level of serum glucose at admission, where high levels of glucose are associated with high mortality [13]. According to Håheim et al., mortality in diabetics is five times higher when compared to non-diabetic patients, and an increase of 1 mmol/L of serum glucose is related to a relative risk of 1.13 [14].

In our study, we found an overall mortality rate of 7.39% and the decomposition in ischemic and hemorrhagic infarction revealed mortality rates of 2.1% and 48.8%, respectively. During the first month of cerebral infarction, mortality is 26% and long-term follow-up, that is, one, two, and three years, reveals cumulative mortality rates of 37%, 46%, and 54%, respectively. In this study, Loor et al. reveal that patients with cerebrovascular disease have a higher risk of dying, which is approximately twice that of the general population, for age and corresponding sex, during the next three years following cerebral infarction [15].

In order to reduce the mortality of patients with cerebrovascular disease at the end of the last century, specialized treatment units (“Stroke Units”) were created. The effect of these treatment units is documented in the work of Jorgensen and collaborators, who studied similar populations, one group under specialized treatment and another under conventional treatment, demonstrating that patients admitted to specialized units have significantly lower mortality (0.34 x 0.74), reduction in fatal cases (0.28 x 0.71), reduction in mortality in six months (0.39 x 0.82), mortality in one year (0.42 x 0.84), and need for follow-up home care (0.38 x 0.98) [16].

In Brazil, stroke was responsible for 10.18% of all deaths in the country in 2009 and the fourth leading cause of years of life lost in 2016 [17]. Although a study in public hospitals in our country has registered a decrease in intra-hospital mortality in recent years [17].

## Conclusions

Our work calls attention to this important health problem, the causes of death in patients with acute CVD and its potential prevention, and confirms the severity of cerebral hemorrhage, with the high mortality rate finding in our country, and the decrease in mortality in patients with cerebral infarction as a result of the implementation of multi-professional care and advances in therapeutic choices. We cannot extrapolate the study to the whole of Brazil, but it has excellent reliability and validity considering our city and most likely the Southern part of Brazil, which is highly homogeneous.
